# Adapting to change: How has COVID-19 affected people’s work and personal goals?

**DOI:** 10.1371/journal.pone.0262195

**Published:** 2022-02-03

**Authors:** Laura M. Vowels, Rachel R. R. Francois-Walcott, Katherine B. Carnelley, Emily L. Checksfield

**Affiliations:** School of Psychology, Life and Environmental Sciences, University of Southampton, Southampton, United Kingdom; Koc University School of Medicine, TURKEY

## Abstract

COVID-19 has had a devastating impact on the global economy and affected millions of people’s work and personal lives across the world. The purpose of the present study was to better understand how individuals’ work and personal goals have been affected by the pandemic and how they have adapted to these changes. We conducted qualitative semi-structured interviews (*n* = 48) and surveyed participants (*n* = 200) weekly for 5 weeks. Both methods revealed similar themes regarding the adaptation and pursuit of goals (social support, handling unpredictable situations, logistics, solving problems creatively, goal postponement, and no changes). Survey responses also showed that most individuals experienced their goals as more difficult (79%; 13% easier; 9% no change) and found that many had had to adapt or postpone their work and personal goals, often due to logistical difficulties. Businesses and governments should do more to help individuals adapt their goals to the new circumstances.

## Introduction

The COVID-19 pandemic has had a devastating impact on the world economy, affecting millions of people’s working lives and careers globally. In 2020, The World Economic Forum [1] forecasted that the pandemic would cause the worst global recession since World War II with global unemployment expected to rise to its highest level since 1965. Indeed, global working hours reduced by 8.8% in the fourth quarter of 2020 (the equivalent of 255 million full-time jobs), and worldwide employment losses increased by 114 million jobs relative to 2019 [2]. In order to curtail the spread of the coronavirus, most countries imposed social distancing measures including working from home, school closures, and avoiding social situations [3]. Therefore, even the people who have been able to keep their jobs during the pandemic are likely to have experienced changes to how they work.

Furthermore, working remotely from home can lead to a gradual blurring of lines between work and family [4]. At the same time as adjusting to working from home, many couples have had to balance childcare and home-schooling responsibilities due to school closures. Because undertaking multiple role transitions can be challenging, the impact of these changes cannot be underestimated [5]. Simultaneously, social distancing measures have meant that individuals have fewer opportunities to replenish their emotional and cognitive resources that have been depleted by work and family demands [6]. This may result in underperforming both at work and in one’s personal life and can result in exhaustion, stress, and burnout [7].

Little empirical evidence exists currently on how the pandemic has affected individuals’ ability to complete tasks and goals at work and in their personal life, and how individuals and families have adapted to changes in circumstances. A poll of 180 employees found work-life balance and productivity had improved whereas work morale and motivation had decreased since the pandemic [8]. Another UK poll of 1,500 business owners and staff found that 53% of respondents believed they or their employees were more productive and had better mental health while remote working [9]. While this research provides some understanding of how the pandemic has affected productivity, it does not address change over time or whether the pandemic has impacted goals beyond work productivity. In the present study, our aim was to add to this literature by studying the impact of the pandemic on goal adaptation over a period of five weeks using a combination of methods (qualitative semi-structured interviews and open-ended survey responses). Specifically, we examined how the pandemic affected individuals’ work and personal goals (e.g., education, health, leisure) and how individuals adapted to the new circumstances.

A number of theories on self-regulation and goal pursuit exist. Examples of these theories include the multiple resource allocation model [10], task goal theory [11], and social-cognitive theory [12]. A common thread among all of these theories is the description of a dynamic and cyclical self-regulation process by which individuals set goals, allocate resources to pursue and maintain these goals, seek feedback from others, evaluate progress, and adjust their goals as needed [13]. In the current research, we would expect to find that participants have regulated and adapted their goals during the pandemic in ways that are reflective of the cyclical self-regulation process described by Vohs and Baumeister [13]. For example, the new changes to people’s work and personal lives will likely have led to a reallocation of resources to maintain current goals, while changing circumstances will have led to the formation of new goals and the alteration or abandonment of others. It is also likely that social support and feedback from others will have become increasingly important for effective goal adaptation and pursuit, due to the new norm for many of working remotely.

Goal adaptation itself can be defined as an individual altering a pre-existing goal and their pursuit of it, particularly in response to a change in circumstances outside of their control [14]. According to goal-setting theory [11], individuals set themselves specific and challenging goals, and then aim to achieve them. However, if an original goal suddenly becomes unattainable, for example, due to a change in circumstances, the individual may be required to adapt their goal and how they continue to pursue it. Goal adaptation is an effective means of self-regulating one’s goals, and can include strategies such as reprioritization, goal postponement, and in the case of unattainable goals, goal disengagement [14].

Adjusting goals (including changing, stopping, or starting goals); reallocating resources across multiple simultaneous demands; and adjusting expectations on progress, may be particularly important during the pandemic, given the changes to people’s work and personal lives. For example, many people are having to adjust how they complete work-related tasks because access to support from colleagues or required technology may not be readily available. Some have lost their employment and have a new goal of applying for jobs. Yet others may have to reallocate resources to particular goals such as housework, childcare, and work given the increased demands across these areas. Given these new challenges that many individuals are facing in the pursuit of their goals, it is important to explore how individuals adapt their goals within the context of this new environment, particularly since the sustained pursuit of these projects has been shown to significantly enhance psychological wellbeing [15, 16].

Previous research into goal pursuit in the context of COVID-19 has explored the impact of the pandemic on the pursuit of personal goals [17]. The study found that, over a ten-day window during the early period of the first lockdown, individuals experienced a significant drop in their self-efficacy beliefs for goal achievement compared to retrospective pre-pandemic ratings. Over a quarter of the participants were no longer pursuing, or were unsure about continuing to pursue, their current goals despite nearly 90% of people reporting that they still cared about their goal. The study provided an important “snapshot” of how COVID-19 has disrupted pursuit of personal goals.

### The current study

Our current research aimed to extend Ritchie et al.’s [17] study by examining how individuals adapted their goal pursuit over a longer period (five weeks) during the initial lockdown. The present research also focused on both the pursuit of personal goals as well as work goals, to shed light on adaptability for both types of goals under the new circumstances. This is particularly important considering the preponderance of individuals working from home during the lockdown, a circumstance which puts their work and personal goals into closer proximity. We also conducted semi-structured interviews to provide further insight into how the pandemic affected goal pursuit and how individuals adapted to the new circumstances. Specifically, we asked participants to report how their goal pursuit had changed as a result of the pandemic and how they had adapted to these changes. Some participants were interviewed about their experiences and some were surveyed weekly over five-weeks. We collected data from individuals currently living with their partner as they were likely to experience work and family conflict and were thus having to adapt their performance across multiple domains (work, family, hobbies).

## Method

### Participants

Survey (n = 200, with an attrition rate of 8.5% at the end of the five weeks) and interview participants (n = 48) had similar demographic characteristics (see Table 1). Participants were 36 years old on average and had been in a relationship for 11 years. The samples were primarily white, heterosexual, and from the United Kingdom. Across both samples, around half the participants were married and half cohabiting, and half of them had children. About 25% of the samples reported that their employment status had changed as a result of the pandemic: seven participants reported that they had been furloughed, 26 had had to stop working, and eight said their workload had reduced. Most participants were now working from home compared to 31% who would normally work from home. Only a minority were keyworkers. Keyworkers are considered critical during the coronavirus pandemic, for example, healthcare, supermarket, and teaching staff.

**Table 1:** Demographic variables.

	Survey (n = 200)		Qualitative (n = 48)	
	m	SD	m	SD
Age	36.5	12.3	36.0	12.9
Relationship length	11.1	9.32	10.4	10.9
	n	%	n	%
Gender				
Woman	105	52.5	33	68.8
Man	93	46.5	15	31.1
Other	2	1.0	0	0.0
Sexual orientation				
Heterosexual	182	91.0	36	76.6
Bisexual	9	4.5	7	14.9
Lesbian/Gay	7	3.5	4	8.5
Other	2	1.0	0	0.0
Relationship status				
Married	102	51.0	26	55.2
Cohabiting	98	49.0	22	46.8
Children				
No	95	47.5	33	70.2
Yes	105	52.5	13	29.8
Ethnicity				
White	184	92.0	41	87.2
Black	5	2.5	1	2.1
Asian	6	3.0	4	8.5
Mixed	2	1.0	1	2.1
Education				
Graduated high school	28	14.0	4	8.5
Some college	38	19.0	4	8.5
Undergraduate	74	37.0	17	36.1
Postgraduate	52	26.0	19	40.4
Other	8	4.0	4	8.5
Employment status				
Employed full-time	121	60.5	21	44.7
Employed part-time	23	11.5	6	12.8
Self-employed	26	13.0	6	12.8
Student	4	2.0	6	12.8
Unemployed	7	3.5	4	8.5
Retired	9	4.5	3	6.4
Employment changed				
No	153	76.5	33	70.2
Yes	47	23.5	14	29.8
Usually work from home				
No	138	69.0	33	70.2
Yes	62	31.0	13	27.7
Country				
UK	119	59.5	32	68.1
USA	17	8.5	4	8.5
Other	64	32.0	12	25.5
Keyworker				
No	166	83.0	44	93.6
Yes	34	17.0	3	6.4
Coronavirus symptoms				
No	179	89.5	39	83.0
Yes	21	10.5	8	17.0

### Procedure

The data were collected as part of a larger concurrent mixed-methods study and the number of study participants were chosen based on the requirements for that study. The data, code, and materials for the project can be found here: https://osf.io/qr7cm/?view_only=365bf35f7ddd45548143b851e10cfcd9. Ethical approval was obtained from the authors’ institutional review board. Participants consented to participate in the study, and they were told that their participation was entirely voluntary and their responses would be kept confidential. Survey data were collected via Prolific, an online participant recruitment website, and qualitative interviews were based on a convenience sample recruited via social media. Participants were eligible for the study if they were 18 years old or above and living with their romantic partner in a country where social distancing measures were in place at the time of the baseline survey. Participants recruited through Prolific received up to £6.70 if they completed all follow-ups. Qualitative interview participants were entered into a raffle to win one of two £30 Amazon vouchers after the first interview and one of two £20 Amazon vouchers after the second interview.

Participants completed a survey weekly for five weeks via a survey platform Qualtrics. Participants also completed a daily diary for the first week but these data are not used in the present report as we did not ask how the goals were being affected on a daily basis. The first survey was completed on 31^st^ March, 2020, which was shortly after many countries had gone under lockdown, and included additional baseline demographic questions. Participants were also asked if they had experienced coronavirus symptoms in the past two weeks at baseline and whether anyone was currently experiencing symptoms at each follow-up. In all surveys, we asked participants questions about their relationship and goals. The questions relevant to this study were: “Please list up to 3 tasks or goals you worked on in the past week.” and “Briefly describe how your goals and how you pursue them have been affected by the pandemic.”

The semi-structured qualitative interviews were conducted via Zoom and audio recorded. Interviews were conducted by the first author. The recordings were then transcribed using an artificial intelligence transcription service and edited by research assistants. The semi-structured interview questions were selected based on the requirements for the larger study which focused on romantic relationships and personal goals during the pandemic. The selection of the questions was based on theory and previous research. We asked participants a range of questions about their relationship and goal pursuit during the pandemic; the following question was the most relevant to the present study: “How has working toward goals and tasks changed as a result of the pandemic?” Participants were prompted further to describe how goal pursuit had gotten better or worse and how they had adapted. We also used data from other questions regarding participants’ relationship where goal pursuit was also mentioned (The full interview guide is provided on the OSF project page). All first interviews were completed between 30^th^ March 2020 and 21^st^ April 2020. Participants recruited via Prolific were given an opportunity to also participate in the qualitative interview. A total of 48 participants completed the first qualitative interview (30 were recruited via social media, 18 via Prolific who participated in both survey and interview parts of the study). We invited participants who had completed the first interview in the first two weeks of the qualitative data collection (*n* = 23) to participate in the follow-up interview to better understand how goal pursuit had changed during the course of the lockdown. Nineteen participants completed the second interview. The initial interviews lasted between 14–49 minutes and second interviews between 7–24 minutes.

### Data analysis plan

The qualitative interviews were analyzed using reflexive thematic analysis [18, 19] and completed using NVivo 12.0. The authors utilized a combination of inductive and deductive approaches to coding by using previous literature and theory to guide coding but allowing for new codes to be created throughout the coding process. The first and second author coded the interviews; both familiarized themselves with the data before creating the initial codes. Codes were then refined iteratively by the two coders and the final themes were agreed jointly. Any disagreements regarding the classification of codes were discussed until 100% agreement was reached. ‘[…]’ was used in the quotes if unnecessary detail was removed or to provide needed additional information in the quoted data provided. Repeated filler words such as ‘like’ and ‘yeah’ were excluded to aid readability. Identifying information was removed.

Content analysis [20] was used to analyze the open-ended questions from survey responses. The codes were created based on the qualitative interviews and were divided into no change, goals more difficult, goals easier, and productivity changed. Two trained coders coded the responses. In order to assess inter-rater reliability, 30% (*n* = 299) of the codes were coded by both coders. The inter-rater reliability (weighted Cohen’s Kappa) between the coders indicated almost perfect alignment for main codes (.85) and substantial alignment for sub-codes (.74; [21]). Any discrepancies or questions in the coding were discussed to ensure consistency among coders.

## Results

### Thematic analysis of interviews

A total of six themes (social support, handling unpredictable situations, logistics, solving problems creatively, goal postponement, and no changes) were identified and are described below with additional representative quotes for each theme presented in Table 2. A total of six sub-themes were identified: four within handling unpredictable situations and two within logistics.

**Table 2:** Themes and subthemes with descriptions and representative quotes for changes in goal adaptation.

Themes	Sub-themes	Description	Quotes
Social Support		Open-minded and accepting of others’ circumstances. This may lead to changing own expectation and behaviour.	I think the main thing about working from home is that I’m removed from kind of office drama. Yeah, because I normally work. It’s a very small office, and we’re kind of on top of each other. And there’s a lot of big personalities and a lot of conflict. And I think it’s actually been very helpful for us to be removed from each other. (#32, W, 36)
			I’m really into sports. I’m a sports enthusiast you could say. It’s actually really challenging to work out alone. I mean, I have music and all that. But still, it’s not the same as going to the gym where there are people (#34, M, 18)
Handling unpredictable situations	Future orientation	A shift in focus to future goals rather than the present.	I’ve had some high paid jobs, high ranking jobs, but I wanted to go self-employed and take this wood-working, joinery course to be able to work for myself. And now that’s just stopped. I don’t know when it will restart. I don’t know if it’s, now that the venture that I do want to do in life I don’t know what to do as far as my future goals and future aims now, because this could be another year before being an actual qualified joiner, so that might not happen now. (#26, M, 40)
			The external market sort of disappeared, and my only option is getting a job internally. So sort of changed the structure of how I’m going about looking at advancing my career, essentially. That’s my main goal that I’m working on at the moment. So just trying to find a place for myself in the company the next six months. (#31, M, 29)
	Self-care and wellbeing	A shift in focus to health-related goals	Just kind of keeping safe and making sure we were set up for a lockdown, there’s challenges more are now around the kind of mental side, staying mentally healthy. (#17, W, 41)
			So, my husband and I have been trying to cut down on alcohol and so with all of the pubs being closed, it’s actually a lot easier for me not to have the incentive to, to be drinking and staying out late. So I’m finding it easier with my health and going to bed earlier. Yeah, sleep hygiene I think has been better. (#14, W, 30)
	Productivity	The ability to successfully work on goals. This may be harder/easier due to motivation or focus	I’m not getting distracted by people coming up to my desk or just general noise. […] I find I’ve been able to, when I’m working on my own and not on meetings, I’ve had much more intense focus than I would normally do. (#17, W, 41)
			We started off really well, but as the month has been going on our motivation for stuff is dropping a little bit. (#41, W, 27)
	Time management	Changes in time management may make it easier/harder to pursue goals	I’m trying to take advantage of the fact that I’m at home on my own and within the headspace to write guidance and comment on policies that I don’t usually have time to do. (#45, W, 27)
			It’s going pretty good but I do find sometimes because I have so much free time now and I’m not used to it because I’ve been working since eight years non-stop and not having holidays. So, it’s a bit overwhelming because I have too much time that I find it hard to concentrate when you’re at home. (#20, W, 29)
Logistics	Physically oriented adaptability	New practical arrangements have been organised for task and goal pursuit.	So, the wife’s in there. So, there’s that bit of organization between ourselves about where we are. Luckily, we’ve got enough rooms in the house ready to disappear. (#6, M, 59)
			My partner mostly works in the living room, and I’ve got the corridor to myself, sometimes it’s just close the door if we need that space when we’re working.” (#13, M, 31).
	Learning new tasks and technologies	Adjusting to new online formats to pursue goals	Lots of kind of getting up to date with technology trying to support other people. So, my goals have changed and my focus is elsewhere and it is harder. (#18, W, 32)
			Sort of working from home systems that my company has not advanced. It just makes it a bit slow, more challenging to do quite a lot of other things. (#31, M, 29)
Solving problems creatively		Creative ideas are developed. This may include forward thinking or use of new and different resources	We’re thinking about different ways to continue the research online instead of doing it in person. (#7, W, 26)
			Rather than doing work for clients, I’m having to think about what I can do that’s useful for the business that isn’t paid work. (#4, W, 46)
Goal postponement		Goals have been cancelled or delayed so goal pursuit has stopped	I’m not able to attend networking events and things like that so it’s just pushed back things by a few months really. (#9, M, 47)
			It does feel almost like you’re driving a very fast car, but suddenly the engine is stalled. And it’s taking a bit of time to kind of readjust to not being as busy or as focused on that goal. (#13, M, 31)
No changes		No changes to goal pursuit identified	To be honest, I can’t think of anything. (#38, M, 33)
			That’s a good question. Is it easier? No (#6, M, 59)

#### Social support

Many participants demonstrated and noted a change in social support due to the pandemic. For most participants this had been a positive change: they reported having increased feelings of supportiveness and understanding which they noted had been reciprocated by others: “everyone [being] a lot more understanding about work being completed […] with delays or needing more time to do things. Just because it’s really difficult to be at work and you have distractions” (#45, W, 30). In turn, many participants who had initially felt stressed managing home life and childcare mentioned this increased feeling of understanding, changed their own and others’ behaviors to being more open-minded, and sought more feedback from others. For example, one participant noted that “there’s been a lot of online support and sort of tips coming from other people when it comes to everything children related. So, that has been really good” (#29, W, 32).

Many participants additionally mentioned the support from their partner had changed as they developed a new working relationship to allow them both to achieve work goals. As such, many participants noted they had received encouragement and motivation from their partner and were also able to provide this encouragement back. For example, “having my husband here when he’s also working on things like college work or a bit of housework, that sort of thing, then doing it at the same time, it just feels nice to have some company and it helps motivation.” (#8, W, 30) Thus, some participants stated working from home was beneficial to their work goals as they were able to have their partner as their colleague.

Conversely, many participants identified that the lack of social interaction had made them feel lonely and had created difficulty in completing work. Such difficulty in reaching work goals during the pandemic, despite having a partner who worked from home or liaising virtually, was typically attributed to the sheer differences in the number of people they were able to interact with. For example, one participant noted they “have come from interacting with 300 people in a day. Now one.” (#38, M, 33) As such, many participants felt isolated and struggled to adapt to working in a less social setting. Additionally, this lack of social interaction went further than participants feeling isolated and alone but for many participants it practically deterred achieving work goals. For example, one participant remarked that “it’s a lot more isolating, because I’m used to working in a shared office and having friends and colleagues around and being able to work alongside the people or be bouncing ideas off each other as we’re working” (#46, W, 31). As such, some participants noted that it was difficult to achieve work goals as they were unable to receive continuous feedback: the virtual team dynamic did not create a suitable environment to truly work with their colleagues thus interfering with goal completion.

#### Handling unpredictable situations

A total of four sub-themes (*future orientation*, *self-care and wellbeing*, *productivity*, *time management*) were identified within this theme. This theme was noted by the majority of participants and highlighted the impact of participants’ feelings on their work goals. Many participants identified an increase in negative feelings due to the unpredictable nature of the pandemic, such as feeling demotivated, stressed, frustrated, and in some cases “feeling like everything was beyond [their] control” (#18, W, 32). Conversely, this unpredictability had been positive for a few participants as they noted feeling more at ease and less pressured as the pandemic had allowed them to “carry on living in the moment and not worry too much about the future or the past” (#43, W, 75).

Behaviorally, positive feelings toward unpredictability led to goal changes in terms of *self-care and wellbeing*, *productivity*, and *time management*. An increase in motivation and focus led some participants to consider self-care and wellbeing as the pandemic had emphasized the importance of health. For example, one participant stated “A lot more prioritization of sort of self-care, don’t completely lose it, goals” (#5, W, 36) had become important. This reprioritization was not limited to self-care and wellbeing but also included participants starting new businesses or courses. For example, one participant said “I have time [to do] lots of things that I didn’t have time for. For example, I started to learn the guitar. The online courses also I’ve been wanting to do for a long time and I’m quite happy I have the time to spend on it now.” (#20, W, 29). Furthermore, participants additionally noted they were more productive as they felt less pressured to complete and pursue tasks; this was caused by a reduction in work interactions and generally feeling more relaxed at home. As such, participants often did not create new goals but the pandemic had allowed participants to refocus and dedicate time to pursuing goals they had previously been unable to.

Conversely, many participants identified negative feelings such as demotivation due to the unpredictable nature of the pandemic which practically impacted productivity and the way in which they handled work stress. Many participants struggled to self-motivate and noted an increase in distractions that made it difficult to focus on goals. For example, one participant said that “I think not having a reason to, like get changed and do the little things makes it a lot more difficult for me mentally to be motivated to do anything.” (#37, W, 19). As such, many participants noted they were less productive on work goals than they had previously been.

While most participants focused on the here and now, some participants had become more *future oriented*: this was evident for participants who identified both positive and negative feelings toward unpredictability. For example, some participants noted they felt that “thinking about what the future is gonna look like is more relevant” (#4, W, 46). Participants thus had to be somewhat flexible with their goals and make them bigger or smaller to cope with the circumstances and prepare for the future. Indeed, goals that had once been placed on the backburner were at times placed at a forefront to allow participants to better prepare for the future. As such, participants began to consider the financial and emotional implications of the pandemic. For example, one participant stated, “So financially, I might have to change a couple of things around until I’m back into work full-time” (#28, M, 28). A change in perspective from the present to the future was particularly notable in the follow-up interviews as participants noted a variety of changes such as pregnancy and job allocation and security. Indeed, some participants noted they had been unable to secure a new job as “there’s less jobs out there and people are less willing to hire” (#12, W, 26). Therefore, many participants were having to reconsider their future job prospects in light of the pandemic. Some participants additionally noted within the follow-up interviews that they considered the future alongside their partner and their partner’s goals. For example, one participant noted the pandemic had “made us think about goals a little bit, kind of future-orientated, both individually and together” (#11, W, 36).

#### Logistics

Two subthemes (*physically oriented adaptability* and *learning new tasks and technologies*) were identified within logistics. Many participants mentioned they had to make new practical arrangements in order to complete their tasks and goals. A new reliance on online formats or balancing homeschooling and work were logistical changes commonly mentioned. Many participants thought these new arrangements to be necessary in pursuing goals, thus they rarely appeared fazed but noted it to be an adjustment as they were “getting used to it and kind of figuring it out” (#5, W, 36). Some participants demonstrated *physically oriented adaptability* regarding work-space and creating a suitable work environment. For example, one participant stated, “What I went ahead and did with the help of my partner was set up. Like I had a desk at home, we didn’t really use it. So, I went ahead and set it up for more effective […] productive use so it’s similar to how my office is.” (#3, W, 26) Indeed, rearranging or creating work-spaces was mentioned by a few participants as being necessary in achieving work tasks and goals. Therefore, many participants commonly mentioned that they were able to continue pursuing goals through practically adapting to their new circumstances. Nonetheless, some participants additionally noted how the implications of working from home slowed down goal achievement. Many participants mentioned they felt frustrated and challenged by inadequate practical arrangements such as “quite slow internet” (#40, M, 33). As such, it was noted to be harder to efficiently complete goals than it had been prior to the pandemic. In such instances, some participants were able to overcome these difficulties and continue completing tasks and work goals whereas for other participants this was not possible, leading to *goal postponement*.

Furthermore, for instances in which participants were able to overcome logistical difficulties this was also achieved through *learning new tasks and technologies* including online exams, podcasts and virtual team software with many commonly noting this adjustment to be “challenging” (#11, W, 36). For example, one participant stated “I’ve had to move my courses online. […] I can’t deliver exams in class settings, so I’ve spent a lot of time trying to develop online, multiple choice tests, which I wouldn’t have used […] that’s been challenging” (#23, W, 49). Furthermore participants additionally noted that the new formats had been challenging and caused disruptions due to external factors such as “a lot of professors [struggled] with the online format” (#37, W, 19). Some participants who were currently studying noted that as they relied on their professor to deliver the material, it made the completion of tasks more difficult when their professor was struggling with the technology. Therefore, learning new tasks and technologies was noted to be disrupting to work goals as the participants themselves struggled or had been impacted by those who struggled.

#### Solving problems creatively

A few participants noted that although the pandemic had created challenges, they had been able to creatively address these and adapt their goals accordingly. This allowed them to ‘think out of the box’ and achieve their work goals in a new manner. For example, one participant noted they “quite like[d] the challenge and use[d] it as an opportunity” (#11, W, 36). Some participants thus put a positive spin on the pandemic and the new opportunities it could create. Few participants explicitly noted they had foreseen and solved potential work-related issues. Nonetheless, one participant stated, “I think the focus of my work has changed quite a lot. Most patients are not wanting therapy, most people are wanting to put on hold. So actually, my focus has been a lot more kind of strategic and planning and preparing for what’s to come. Because I think, in community mental health, we’re getting a calm before the storm” (#18, W, 32). However, preventing the occurrence of problems was at times implicitly mentioned by participants in teaching professions who noted that they had to switch to online formats for learning and exams. In this regard, an overlap was present between solving problems creatively and learning new tasks and technologies.

#### Goal postponement

About half the participants mentioned that their goals had to be postponed or cancelled. Most noted this was due to factors outside of their control such as being unable to go on holiday, an inability to pursue work goals, or because of uncertainty about how long the pandemic is going to last and what the world will look like post-pandemic. As such, this theme somewhat overlapped with logistics as some participants noted where it was not possible to complete goals from home, goals were postponed. Some participants were unable to find jobs with one participant noting, “there aren’t many vacancies right now… I’ve received emails saying that they’ve closed so they’re no longer considering applicants or that they’re closing the vacancy” (#12, W, 26). Some participants found this postponement led to changes at work and found that “work is a lot less busy” (#13, M, 31) and they were unable to complete particular tasks and goals.

#### No changes

While almost all participants described that their goal pursuit had changed in some form, a few participants noted no change to goal pursuit regarding the ease of completing goals or creation of goals. For example, one participant said “I think it’s the same it’s just a strange feeling working from home but it’s not harder” (#10, M, 42) and another stated that “nothing much has changed” (#30, W, 39).

### Content analysis of survey responses

We used the themes from the qualitative interviews to guide coding of the open-ended responses. There was not much variation between the codes across the five weeks. Therefore, instead of presenting the responses for each week, we report the aggregate scores across the five weeks (A breakdown of results by week can be found as supplemental material on the OSF project page). The sample of 200 participants provided responses up to five times over the study period. This resulted in a total of 948 responses that were coded into the main codes, with a minority of the responses not being codable (n = 47, 5.0%). The codes were first divided into three categories based on whether goals had become *more difficult* (*n* = 880, 78.64%), *easier* (*n* = 142, 12.69%), or *had not changed* (*n* = 97, 8.67%). The responses were further divided into five specific codes. Many participants reported that goals had become more difficult due to *logistics* (*n* = 276, 29.27%), decline in *productivity* (*n* = 243, 25.77%), and difficulties with *time management* (*n* = 60, 6.36%). Some participants reported that they had *adapted their goals* in some way (*n* = 170, 18.03%) or they had had to *postpone or stop their goal pursuit* (*n* = 80, 8.48%). The only way in which participants reported their goals had become easier was due to having *more time* to pursue goals (*n* = 114, 12.09%). Similar themes were found within the specific codes as in the qualitative interviews. Adapting goals included elements from interpersonal adaptability, handling unpredictable situations, and solving problems creatively but these were difficult to differentiate in the open-ended responses.

## Discussion

The COVID-19 pandemic has affected people’s working and personal lives across the world with millions of people losing their jobs and many more facing significant challenges [22]. In the present study, our aim was to understand how the pandemic had affected individuals’ work and personal goals and how individuals have adapted their goal pursuit to match the challenging circumstances over time. We examined our research questions using two different methods: semi-structured interviews and open-ended survey responses over five weeks. Using multiple methods allowed us to gather in-depth information into how individuals had adapted their goal pursuit and to quantify any changes over time. With the pandemic being an unprecedented event, a more nuanced understanding of the participants’ experiences was especially important, and since prior research into COVID-19 and goal pursuit [17] has only employed the use of survey responses, the inclusion of semi-structured interviews allowed for a more in-depth exploration of the phenomenon.

Previous research has stated that in order to cope with new circumstances, individuals and organizations must be able to adapt quickly to respond to changes in demands [23, 24]. Nearly 80% of the survey participants and the majority of interview participants reported that their goal pursuit had become more difficult. For example, in line with early research into the pandemic [8, 9], a quarter of the participants noted changes in motivation that caused them to become less productive, including having more distractions and a lack of motivation. However, many participants had been able to successfully adapt some of their goals.

We found that logistical challenges were the most commonly reported issue affecting goals during the pandemic. Most participants noted that they had been adapting to these challenges through physically creating workspaces or learning new technologies. A few participants noted technology such as poor internet had made goal progress harder with some additionally identifying their company technology was not sufficient. Many participants also identified perception of social support to have changed, as they were more understanding and supportive of their colleagues and had such feelings reciprocated. Participants who were unable to receive sufficient contact with their colleagues noted they felt lonely and found it practically difficult to complete work without feedback from others. This evidence reinforces the importance of social support in pursuing goals [25, 26]. Problem solving creatively was rarely mentioned: it was however identified by those in teaching professions who were required to quickly adapt to provide sufficient resources to students.

While most participants reported that goal pursuit had become more difficult, around 13% of the survey participants as well as some interview participants reported that goal pursuit had become easier, usually because they had more time to work on goals. Some participants had also altered their focus from work-related tasks to pursuit of self-care and hobbies. A minority of the participants said that there was no change in goal pursuit, which may reflect the fact that some participants already worked from home before lockdown or that they engaged in goal pursuits that were possible to do even during lockdown (e.g., going on walks, eating healthily). Furthermore, we did not find evidence of change over time. Instead, most participants reported that they experienced similar challenges to goals over the course of the five weeks. Some participants did mention a shift to more future-related goals in the second interviews than at the start of lockdown.

The themes identified within this study are broadly similar to those reported in Ritchie et al.’s [17] study. In particular, awareness of the importance of social support, future-oriented planning, and an enhanced focus on self-care were found in both papers to be features associated with continued goal pursuit during the pandemic. This suggests that these strategies have been important during the pandemic for enabling continued goal pursuit. Our findings build on Ritchie et al.’s [17] in that we identified themes, problems, and strategies specific to the pursuit of work-related goals and work-life interactions in addition to solely personal goals. For example, physical logistics was identified as a major barrier to effectively working from home, and the role of partners and colleagues was emphasized in supporting participants to adapt to new ways of working during the pandemic.

Regarding personal goals, the lockdown saw many individuals create new goals surrounding self-care and wellbeing, perhaps due to an increased awareness of health from the pandemic. Meanwhile, other pre-existing personal goals, which had previously been superseded in priority by work goals, were now able to be pursued during an increased amount of free time. The exploration of both work and personal goals during the lockdown showed that the line between the two types of goals became blurred: new personal goals such as homeschooling and childcare became prerequisites for being able to complete work goals, and reduced time spent on pursuing work goals meant increased time available to pursue new personal goals.

Overall, while COVID-19 has impacted everyone, some participants were better able to effectively adapt their goals to the situation, while others have been forced to postpone and even abandon their goals. These findings are consistent with previous research on self-regulation and goal pursuit, with the processes used by participants to adapt their goals being reflective of Vohs and Baumeister’s [13] description of dynamic and cyclical self-regulation of goals. For example, goal pursuit was maintained for some by reallocating resources in the form of rearranging the home to become a workspace, while many have sought increased support and feedback on their goals from their partners and colleagues. Where further goals were evaluated as unfeasible due to the changing circumstances, they were often postponed or abandoned. Together this creates a picture of how individuals have effectively regulated their goal pursuit during the pandemic.

### Limitations and future research

The present study had several strengths. First, we combined two different methodologies: semi-structured interviews and open-ended survey responses. By combining these methods, the study benefited from the generalizability of a larger and more representative dataset and the nuanced and detailed description of participants’ experiences in the interviews. Participants also began completing the surveys from the beginning of many countries’ stay-at-home orders effectively capturing the first month under lockdown, arguably a time that demanded a great deal of adaptation.

However, the study also had some limitations and results should be interpreted with these in mind. Participants were recruited via Prolific hence we potentially captured data from those who had more time available during the pandemic. Short-term and long-term impact of the pandemic may vary [27]. However, our study focused on the first five weeks only and did not show change during this period. Moreover, the current study did not address whether successful adaptation in goal pursuit was associated with better outcomes (e.g., higher job performance) long-term. Therefore, future research is needed to investigate the long-term impacts of the pandemic on goal outcomes. Furthermore, while we examined how goals were affected over time, we did not ask how different types of goals (e.g., work or personal goals) may have been affected differently. It is possible that, for example, individuals’ work-related goals have been negatively affected by the pandemic whereas leisure goals may have been positively affected due to having more leisure time. This idea was largely consistent with the qualitative interviews but future research is needed to examine quantitatively whether this is indeed the case on average. It is also possible that some of the changes to goal pursuit were due to factors other than the pandemic (e.g., poor goal-setting skills, job security, mental health issues) even though participants had identified the pandemic as the reason for the changes. Furthermore, due to the speed with which the virus spread in the first few months and its unprecedented nature, it was not possible to gather pre-pandemic data from participants. We began data collection in the first few weeks of most countries’ lockdowns but the reports of how participants’ goals had changed and how they had adapted rely on participants’ retrospective accounts only. There may be ways in which it is possible to investigate how goals have been affected using available data from before, during, and after the pandemic such as work output, student grades, or census data. These possibilities should be explored in future studies to further understand the scale of the impact the pandemic has had on goals.

### Practical implications

Many participants in the present study highlighted that they had had to adapt or postpone their goals due to logistical difficulties. Some of these difficulties may have been unavoidable due to widespread business closures and orders to work from home. However, there may be other difficulties that could be mitigated by employers and governments especially as stay-at-home orders ease. More employees are set to continue working from home (37%) once the pandemic is over than they were pre-pandemic (18%; [28]) As such, it is important companies address the limitations around technology such as through ensuring that their employees have adequate equipment and broadband as well as access to technological support when working from home. Workplaces should also offer guidance and training on how to adapt to the new working conditions. Additionally, as people attempt to look beyond the pandemic and become future-oriented, an increase in long-term funding or resources may be necessary. Many participants also reported that they had less access to social support and colleagues at work which made them feel more isolated. As organizations adapt to social distancing measures, they should also invest in a virtual working environment or smaller work bubbles (i.e., small number of people working together with the same people) that allow for greater connection between team members. While the results are especially useful during the pandemic, the study has implications for other stressful situations (e.g., the evolution of organizational structures and processes, economic and political instability, and technological advances) [29] that occur more frequently that force individuals to adapt their goal pursuit. Preparing for these eventualities in advance can help mitigate their impact.

## Conclusion

We found that most individuals experienced their goals as more difficult and had had to adapt or postpone many work and personal goals. For many participants, difficulty in goal pursuit was due to logistics and lack of social support. These difficulties could be addressed through further support by employers which would alleviate the additional stress and frustration participants noted. Thus, businesses and governments should do more to help individuals adapt their goals to the new circumstances.
